# Synergistic Antibacterial Effects of Plant Extracts and Essential Oils Against Drug-Resistant Bacteria of Clinical Interest

**DOI:** 10.3390/pathogens14040348

**Published:** 2025-04-04

**Authors:** Hoda Helene Shahin, Moomen Baroudi, Fouad Dabboussi, Bassel Ismail, Rayane Salma, Marwan Osman, Khaled El Omari

**Affiliations:** 1 Laboratoire Microbiologie Santé et Environnement (LMSE), Doctoral School of Sciences and Technology, Faculty of Public Health, Lebanese University, Tripoli 1300, Lebanon; hoda.shahin.1@ul.edu.lb (H.H.S.);; 2 Water and Environment Sciences Laboratory-EDST, Lebanese University, Tripoli 1300, Lebanon; mbaroudi@ul.edu.lb; 3 College of Health and Medical Technologies, Alayen Iraqi University (AUIQ), Nasiriyah 64001, Iraq; bassel.adel@alayen.edu.iq; 4 Quality Control Center Laboratories at the Chamber of Commerce, Industry & Agriculture of Tripoli & North Lebanon, Tripoli 1300, Lebanon; 5 Department of Neurosurgery, Yale University School of Medicine, New Haven, CT 06510, USA

**Keywords:** antibacterial activity, crude extract, essential oil, synergism, antimicrobial resistance, bacterial resistance

## Abstract

Infectious diseases, the second leading cause of death worldwide, have traditionally been treated with antimicrobials. However, the emergence of drug-resistant microorganisms has driven the need for alternative therapies. This study aimed to assess the antibacterial efficacy of *Capparis spinosa* crude extracts and five essential oils (EOs) derived from *Salvia officinalis*, *Eucalyptus globulus*, *Micromeria barbata*, *Origanum vulgare*, and *Juniperus excelsa*. The EOs were extracted using hydro-distillation, and *C. spinosa* extracts were obtained using ethanol and acetone solvents. Microdilution assays revealed that *O. vulgare* EO exhibited the strongest activity against *Listeria monocytogenes*, *Escherichia coli*, *Salmonella* spp., and *Brucella melitensis*, while *C. spinosa* demonstrated significant antibacterial effects against *L. monocytogenes* and notable inhibition of *Pseudomonas aeruginosa*. The combination of EOs with antibiotics, including *M. barbata*, *J. excelsa*, *S. officinalis*, and *E. globulus*, enhanced the efficacy of the antibiotics against recalcitrant bacterial strains. The synergistic effects were evaluated through Fractional Inhibitory Concentration Index (FICI) analysis. These findings confirm that the antibacterial efficacy observed in the tested EOs, especially when used in synergy with antibiotics, offers a promising therapeutic strategy to combat antimicrobial resistance.

## 1. Introduction

The discovery of penicillin started a golden age in antibiotic research and development, which revolutionized modern medicine and significantly extended the lifespan of humans [1]. Antibiotics mainly function by suppressing bacterial cell wall construction, altering membrane integrity, inhibiting nucleic acid and protein production, and interfering with metabolic processes [2].

The overuse of antibiotics in various sectors, such as medicine, agriculture, and the animal food industry, has led to bacterial resistance under selective pressure, representing a new paradigm in pathogenesis, transmission, and resistance [3,4]. This resistance initially developed in staphylococci, streptococci, and gonococci, following the release of the first commercial antibiotic, penicillin, in 1941, and penicillin-resistant *Staphylococcus aureus* emerged a year later, in 1942 [5]. Nowadays, addressing patients infected with multidrug-resistant (MDR), extensively drug-resistant (XDR), or pandrug-resistant (PDR) bacteria poses a serious challenge [6]. As a result, pharmaceutical companies face financial and regulatory challenges, and significant investments in scientific research were allocated to combat the resistant bacteria [5].

*Acinetobacter baumannii*, commonly found in hospital environments, particularly in intensive care units, is a major cause of ventilator-associated pneumonia, bloodstream infections, and wound infections, with many strains exhibiting resistance to colistin and other antibiotics [7]. Similarly, XDR *Escherichia coli*, which is linked to severe urinary tract infections, often exhibits resistance to carbapenems, leading to treatment failures and increased mortality in critically ill patients [8]. Both pathogens pose significant challenges to effective infection management in healthcare settings [9].

*Campylobacter coli* has developed increasing resistance to antibiotics, particularly those that are typically used as first-line treatments for campylobacteriosis [10]. These resistance patterns complicate treatment strategies for infections, particularly in the healthcare setting, where *C. coli* can lead to severe gastrointestinal and systemic infections [11].

*Pseudomonas aeruginosa* is known for acquiring resistance to β-lactams, including carbapenems, ceftazidime, and piperacillin-tazobactam, particularly in healthcare settings [12]. It is responsible for severe infections such as those associated with burns, catheterization, and neutropenia, leading to septicemia, urinary tract infections, and bacteremia [13]. *P. aeruginosa* represents approximately 7.3% of all healthcare-associated infections [14].

The 2021–2022 European Union report highlighted the growing issue of antibiotic resistance in *Salmonella*, particularly in poultry-associated strains [15]. Resistance to critical antibiotics, such as third-generation cephalosporins, is increasing, with extended-spectrum β-lactamase (ESBL)-producing strains becoming more widespread. This rise in resistance presents a significant challenge to both healthcare and food safety, as these strains complicate treatment options and elevate the risk of foodborne outbreaks [15].

The global priority pathogens list published by the World Health Organization (WHO) classified antibiotic-resistant bacteria into critical, high, and medium priority tiers, emphasizing the immediate requirement for research and developing new treatment options [16]. New strategies are being implemented to overcome antimicrobial resistance, some of which focus on the antibacterial effect of essential oils (EOs) and their synergism with antibiotics [17].

EOs are aromatic and volatile liquids derived from plant materials such as bulbs, roots, bark, leaves, seeds, peels, fruits, and wood that can give plants their unique scent, taste, or both. EOs are hydrophobic, soluble in alcohol, non-polar or weakly polar solvents, waxes, and oils, but only partly soluble in water, many of which are uncolored or pale yellow [18,19]. EOs have been used for centuries in medicine, cosmetics, perfumes, and aromatherapy and have been applied to food as part of spices or herbs [20]. Recent research has demonstrated their potential as antimicrobial agents, with studies showing their efficacy against foodborne pathogens such as *Listeria monocytogenes* and *Salmonella* Typhimurium [21]. Additionally, certain components, such as thymol and carvacrol from oregano oil, have exhibited significant antibacterial properties by disrupting bacterial cell membranes [22], where pathogens cannot simply develop resistance to the EO because of its complex mixture of multiple compounds [23]. EOs influence bacterial cells through many pathways, including targeting cell membrane phospholipids, affecting enzyme processes, damaging genetic material, and producing fatty acid hydroperoxide triggered by the oxygenation of unsaturated fatty acids [4,24].

The floristic richness in Lebanon is estimated to be around 2600 plant species, with endemic plants accounting for 12% of the total, all within a relatively small area of 10,452 square kilometers [25].

Lebanese plants, including *Origanum vulgare* (Linnaeus, 1753), *Micromeria* spp. (Linnaeus, 1753), *Salvia officinalis* (Linnaeus, 1753), *Eucalyptus globulus* (L’Héritier 1788), and *Juniperus excelsa* (Pallas, 1771), are valued for their EOs with antimicrobial properties. *Capparis spinosa* (Linnaeus, 1753), native to the Mediterranean and present in Lebanon, demonstrates a wide range of bioactivities, such as antibacterial, antifungal, antioxidant, anti-inflammatory, and anticarcinogenic effects [26,27]. *O. vulgare* has been used since ancient times as a food seasoning and effectively targets foodborne pathogens [28], while *Micromeria* species are particularly effective against Gram-positive bacteria and exhibit significant free radical scavenging properties [29]. *S. officinalis* is widely used in traditional medicine, especially for women’s health, and has attracted attention for its therapeutic potential [30,31]. In Tunisian folk medicine, inhalation of *E. globulus* EO has been traditionally used to manage respiratory disorders such as pharyngitis, bronchitis, and sinusitis [32]. *J. excelsa* offers antimicrobial and antioxidant benefits, primarily used in traditional medicine to address conditions like cough, dysmenorrhea, jaundice, tuberculosis, bronchitis, and colds, as well as to induce menstruation and expel the fetus [33].

The present study aims to evaluate the antibacterial effect of *O. vulgare*, *S. officinalis*, *E. globulus*, *J. excelsa* EOs, *and Capparis spinosa* crude extracts against *L. monocytogenes*, XDR *P. aeruginosa*, XDR *E. coli*, ESBL-producing *Salmonella* spp., and *Brucella melitensis.* Also, the synergistic antibacterial effect of *J. excelsa*, *S. officinalis*, *Micromeria barbata*, and *E. globulus* EOs associated with commercial antibiotics against *E. coli*, *P. aeruginosa*, XDR *A. baumannii* strains, and MDR *C. coli* strains is evaluated. This assessment could offer a novel approach to enhancing the efficacy of existing antimicrobial agents, providing a promising alternative to combat resistant infections.

## 2. Materials and Methods

### 2.1. Preparation and Extraction of Plant Materials

Plant materials of *Salvia officinalis*, *E. globulus*, *Micromeria barbata*, *Origanum vulgare*, *Juniperus excelsa*, and *Capparis spinosa* were obtained through wild harvest from the North Lebanon Region. Specifically, *M. barbata* and *J. excelsa* were harvested in Denniyeh, *O. vulgare* and *C. spinosa* in Abu Samra, during August, *S. officinalis*, and *E. globulus* in Tripoli during September. Whole plant parts were used for essential oil (EO) extraction, except for *C. spinosa*, where only the leaves were utilized. The plant materials were air-dried at room temperature (28 °C) for approximately 4 days, and EOs were extracted using the hydro-distillation method with a stainless steel hydrodistillator at the Chamber of Commerce, Industry, and Agriculture in Tripoli. After weighing, each batch of plant samples was submerged in 18 L of clean water heated to 95 °C for 4 h and separated into aqueous and oil-rich layers. The oil was collected using a separating funnel, stored in opaque glass bottles, and refrigerated at 4 °C until analysis [34]. The yield of EOs from *J. excelsa*, *M. barbata*, and *E. globulus* was 2% when extracted from 2000 g of the plants on a moisture-free basis, while *O. vulgare* yielded 0.6%, and *S. officinalis* yielded 1.2% from the same weight of whole plant material. Leaves from *C. spinosa* were pulverized into a coarse powder using a pestle and mortar. For extraction, 10 g of the powder was mixed with 100 mL of 100% ethanol or 200 mL of 80% acetone [35,36]. The mixtures were kept at 4 °C for three days. The extracts were filtered through Whatman No.1 filter paper. Solvents were removed at 40 °C to obtain *C. spinosa* acetonic extracts (*C. spinosa*-ac) and *C. spinosa* ethanolic extracts (*C. spinosa*-etOH).

### 2.2. Antibiotics

The selected antibiotics were gentamicin (GEN, Panpharma, Brombach, Germany), tetracycline (TET, Pharmadex S.A.L, Kahaleh, Lebanon), ciprofloxacin (CIP, Ladinin200, Athens, Greece), levofloxacin (LEV), amikacin (AMI), cefepime (CEF), and amoxicillin–clavulanic acid (AMC), all obtained from SIGMA. Antibiotics were used as recommended by the Clinical and Laboratory Standards Institute (CLSI) [37].

### 2.3. Bacterial Strains

The bacterial strains examined, as listed in Table 1, were obtained from the Microbial Collection of the Lebanese University (CMUL) at the Laboratoire Microbiologie Santé et Environnement (LMSE). These strains were selected based on their clinical significance and antimicrobial resistance profiles. The collection includes MDR, XDR, and ESBL-producing strains, which are frequently implicated in hospital-acquired infections and pose major treatment challenges.

The inoculum was prepared from a pure culture incubated for 24 h on Mueller–Hinton II agar medium (Bio-Rad, Marnes-la-Coquette, France) for the bacterial strains. The microorganisms were then suspended in sterile brain heart infusion (BHI) broth (Scharlau, Barcelona, Spain) supplemented with 0.2% agar (Bio-Rad, Marnes-la-Coquette, France) to achieve turbidity levels approximating 0.5 and 1 McFarland.

Antimicrobial susceptibility was assessed using phenotypic testing on Mueller–Hinton II agar, with results interpreted according to EUCAST guidelines 2023 (https://www.eucast.org/clinical_breakpoints, accessed on 14 March 2025).

### 2.4. Determination of Minimum Inhibitory Concentration (MIC)

**Figure 1:**
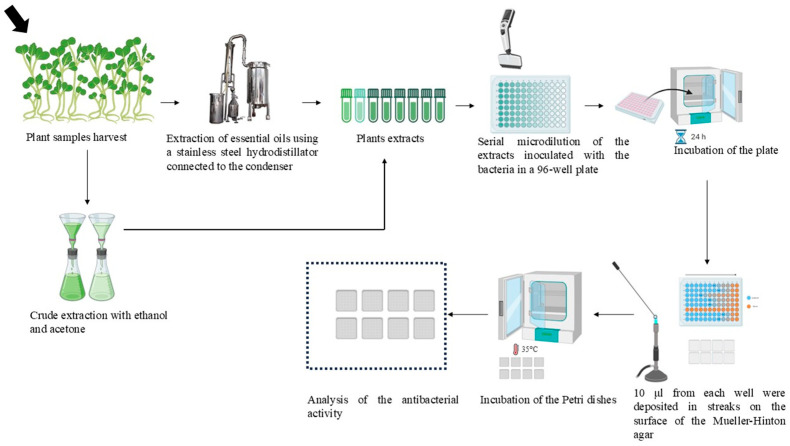
Extraction methods of essential oils, Capparis spinosa crude extracts, and evaluation of the antibacterial activity by microdilution.

MIC of EOs, *C. spinosa*-ac, and *C. spinosa*-etOH were determined by serial microdilution in 96-well microplates, as described by Diniz et al. Also, 200 µL of the broth was pipetted in the well of the first row as a negative control (A1), and 200 µL of bacterial inoculum of 10^6^ CFU/mL with BHI was inoculated in the well of the second row as a positive control (A2) [38]. EOs and extracts were prepared using a diluent containing 0.5% TWEEN 80 (Fluka, Darmstadt, Germany). Serial dilutions of 1/2, 1/4, 1/8, 1/16, 1/32, 1/64, 1/128, 1/256, and 1/1024 resulted in final concentrations ranging from 50% *v*/*v* to 0.10% *v*/*v*. The plates were incubated at 35 °C for 18–24 h. At last, 10 μL from each well were deposited in streaks on the surface of the Mueller–Hinton agar. The Petri dishes were incubated at 35 °C for 18 h before analysis, as shown in Figure 1. The experiment was conducted three times independently to ensure the reliability of the results.

### 2.5. Synergistic Effect of Essential Oils and Antibiotics

The synergistic effect of EOs and antibiotics was analyzed using a serial twofold microdilution method in 96-well microplates to determine the MIC [39]. Antibiotics were tested at the following concentration ranges: AMI and AMC, 8 to 2 mg/L; LEV, 1 to 0.25 mg/L; GEN and TET, 2 to 0.5 mg/L; CIP, 0.5 to 0.125 mg/L.

For EOs, concentrations were prepared using a stepwise dilution from 45% *v*/*v* to 25% *v*/*v*. Each well contained a total volume of 200 µL, consisting of 100 µL of bacterial inoculum (10⁶ CFU/mL), EO, and an antibiotic. The EO volume was adjusted according to its concentration, such that for 45% EO, the well contained 90 µL of EO and 10 µL of antibiotic, while proportional adjustments were applied for lower EO concentrations.

Plates were incubated at 35 °C for 18–24 h. Following incubation, 10 µL from each well was streaked onto Mueller–Hinton agar plates and incubated at 35 °C for 18 h. The experiment was conducted three times independently to ensure the reliability of the results.

### 2.6. Fractional Inhibitory Concentration Index (FICI) Analysis

The FICI of EOs combined with antibiotics was calculated to evaluate their interactions against resistant bacterial strains. The FICI was calculated using the following formula: FICI = (MIC of EO in combination/MIC of EO alone) + (MIC of antibiotic in combination/MIC of antibiotic alone) [40]. The highest tested concentration was considered MIC when the EO showed no activity [41]. The concentrations of EOs and antibiotics were expressed in mg/L for consistency, with FICI values interpreted as follows: FICI ≤ 0.50 indicates synergy, 0.50 < FICI ≤ 1.00 is additive, 1.00 < FICI ≤ 4.00 indicates indifference, and FICI > 4.00 suggests antagonism [42].

### 2.7. Statistical Analysis

Statistical analyses were performed using IBM SPSS software, version 26. The mean and standard deviation were analyzed by mean comparison to determine the most effective EO and crude extract, as well as the most susceptible bacterial strains. The Kruskal–Wallis test, a non-parametric method, was used to assess the significance of differences between the EOs and bacterial strains. Since SPSS version 26 does not include pairwise post-hoc comparisons for Kruskal–Wallis, the Mann–Whitney U test was applied for pairwise comparisons. All tests were two-sided, with a significance level set at α = 0.05.

### 2.8. Chemical Analysis

The chemical composition and concentrations of the component in the EOs of *M. barbata*, *J. excelsa*, *O. vulgare*, and *E. globulus* were determined through capillary column GC-MS (Shimadzu QP 2010), with injection conducted in splitless mode [29].

## 3. Results

The results obtained from the three independent repetitions were consistent, with no significant variations observed among them.

### 3.1. Antibacterial Activity of the Essential Oils

The results of the microdilution of the EOs against *E. coli* CMUL 260, *E. coli* CMUL 096, *P. aeruginosa* CMUL 122, *Salmonella* spp. CMUL 216, *L. monocytogenes* AL004, and *B. melitensis* CMUL 05 are shown in Table 2.

*S. officinalis* essential oil showed antibacterial activity against *E. coli*, *Salmonella* spp., *L. monocytogenes*, and *P. aeruginosa*. However, *E. coli* CMUL 096 showed the highest sensitivity to the EO.

*E. globulus* EO exhibited antibacterial activity against *E. coli*, *Salmonella* spp., *L. monocytogenes*, *P. aeruginosa*, and *B. melitensis* strains. Among them, *L. monocytogenes* showed the highest sensitivity, with a 0.78% *v*/*v* MIC value, while the Gram-negative bacteria displayed a MIC range of 1.56% *v*/*v* to 50% *v*/*v*. Additionally, *E. coli* CMUL 260 was more sensitive to the EO than *E. coli* CMUL 096.

The antibacterial activity of *O. vulgare* EO was also observed against the bacterial strains. The EO inhibited *E. coli* CMUL 260, *Salmonella* spp., and *L. monocytogenes* at a 0.10% *v*/*v* MIC value. Notably, *E. coli* CMUL 260 showed higher sensitivity than *E. coli* CMUL 096.

*J. excelsa* EO demonstrated antibacterial activity against *B. melitensis* and *E. coli* CMUL 096, with *B. melitensis* showing sensitivity to the EO at a MIC value of 6.25% *v*/*v*.

Kruskal–Wallis tests revealed statistically significant differences in the antibacterial activities of the EOs (*p*-value = 0.035). *O. vulgare* EO was the most effective, exhibiting the lowest mean percentage of activity (4.28%) and minimal variability (standard deviation = 10.15%), followed by *E. globulus* EO, which showed a significant difference (*p*-value = 0.029). *S. officinalis* EO exhibited significantly lower activity than *O. vulgare* (*p*-value = 0.019). *J. excelsa* EO was the least effective, showing markedly lower activity than *O. vulgare* EO, though the difference was not statistically significant (*p*-value = 0.086).

There were no statistically significant differences in the susceptibility of the bacterial strains (*p*-value = 0.255). The highest susceptibility was observed in *E. coli* CMUL 260, with a minimal mean percentage (mean = 2.64%) and minimal variability (standard deviation = 3.21), indicating highly consistent results. *Salmonella* spp. also showed strong susceptibility, though the difference compared to *E. coli* CMUL 260 was not significant (*p*-value = 0.822).

When comparing the two *E. coli* strains, *E. coli* CMUL 096 exhibited markedly lower susceptibility than *E. coli* CMUL 260, but this difference was also not statistically significant (*p*-value = 0.289).

*L. monocytogenes* exhibited moderate susceptibility (*p*-value = 1.00), while *B. melitensis* showed lower susceptibility, with no statistically significant difference (*p*-value = 0.212) compared to *E. coli* CMUL 260. Finally, *P. aeruginosa* exhibited the least susceptibility to the EOs, with a statistically significant difference in effectiveness compared to *E. coli* CMUL 260 (*p*-value = 0.046), indicating its higher resistance relative to the other bacterial strains.

### 3.2. Antibacterial Activity of the Crude Extracts

**Figure 2:**
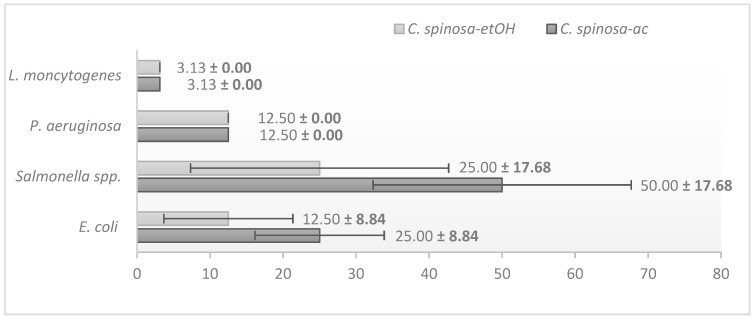
Minimum inhibitory concentration (%) of *Capparis spinosa* crude extracts, showing the highest antibacterial activity against *Listeria monocytogenes*, as indicated by the lowest MIC value. Error bars represent the mean ± standard deviation (%) for the susceptibility of bacterial strains to the crude extracts.

*C. spinosa* acetonic extracts (*C. spinosa*-ac) and *C. spinosa* ethanolic extracts (*C. spinosa*-etOH) were prepared and evaluated against *E. coli* CMUL 260, *Salmonella* spp., *L. monocytogenes*, and *P. aeruginosa* CMUL 122 strains. *L. monocytogenes* exhibited the highest sensitivity, requiring the lowest concentration for inhibition for both extracts (mean = 3.13%, standard deviation < 0.001). *P. aeruginosa* also showed high susceptibility, with a 12.50% *v*/*v* MIC value (mean = 12.50%), while *E. coli* demonstrated moderate susceptibility (mean = 18.75%). *Salmonella* spp. was the least susceptible, requiring the highest concentration for inhibition (mean = 37.50%), as shown in Figure 2. There were no statistically significant differences in the susceptibility of the bacterial strains (*p*-value = 0.101). Notably, *C. spinosa*-EtOH (mean = 13.28%) exhibited greater overall efficacy compared to *C. spinosa*-ac (mean = 22.66%), although the difference was not statistically significant (*p*-value = 0.549). This result highlights the potential of *C. spinosa*-EtOH as a highly effective extract, particularly against *L. monocytogenes* and *P. aeruginosa*.

### 3.3. Effect of Associating Levofloxacin and Amikacin with M. barbata, J. excelsa, and E. globulus

The control of the three EOs against XDR *A. baumannii* and *P. aeruginosa* CMUL 122 strains showed bacterial growth, except for *M. barbata*, which showed antibacterial activity against *A. baumannii* till 0.20% *v*/*v* MIC. So, its tested concentrations were 0.39% *v*/*v*, 0.20% *v*/*v*, and 0.10% *v/v.*

Total inhibition of *P. aeruginosa* colonies with 45% *v*/*v* of *M. barbata* EO and each 2, 4, 8 mg/L of AMI, 35% *v*/*v* of the EO combined with each 4 and 8 mg/L of AMI. The significant antibacterial activity was for 4 mg/L of AMI associated with 35% *v*/*v* of *M. barbata* EO. FICI results showed that this combination had an additive action against the bacterial strain (FICI = 0.85), as shown in Table 3.

Total inhibition of *A. baumannii* colonies was observed with all combinations of 25, 35, and 45% *v*/*v* of J. excelsa EO with 2, 4, and 8 mg/L of AMI, and 0.25, 0.5, and 1 mg/L of LEV. The highest antibacterial activity was noted when 25% *v*/*v* of *J. escelsa* was combined with each 2 mg/L AMI and 0.25 mg/L LEV. FICI results showed that these combinations synergized against the bacterial strain (FICI = 0.50). Same for all *E. globulus* concentrations combined with LEV dilutions, where 25% *v*/*v* of the oil with 0.25 mg/L of the antibiotic showed optimal inhibition and synergistic action (FICI = 0.50). However, only 2 mg/L of AMI with all *E. globulus* concentrations, especially 25% *v*/*v*, showed total inhibition, with synergistic activity (FICI = 0.50). *A. baumannii* colonies inhibition for the following associations: 1 mg/L of LEV and 0.10% *v*/*v*, 0.20% *v*/*v*, and 0.39% *v*/*v* of *M. barbata* EO, 0.5 mg/L LEV and 0.10% *v*/*v* and 0.39% *v*/*v* of *M. barbata* EO. The optimal bacterial inhibition and synergistic action were marked when combining 0.25 mg/L of LEV with 0.10% *v*/*v* of *M. barbata* EO (FICI = 0.50). Moreover, additive actions were noted against XDR *A. baumannii*, as demonstrated in Table 4.

### 3.4. Effect of Associating AMC with J. excelsa, M. barbata, and E. globulus

A control of *J. excelsa*, *M. barbata*, *and E. globulus* EOs against XDR *E. coli* CMUL 260 showed zero antibacterial activity.

Antibacterial activity was observed when both 35% *v*/*v* and 45% *v*/*v* of *E. globulus* EO dilutions were associated with 2, 4, and 8 mg/L of AMC, also with 25% *v*/*v* of the EO combined with 8 mg/L of AMC.

Both 45% *v*/*v* and 25% *v*/*v* concentrations of *M. barbata* EO with 2 mg/L AMC showed an optimal inhibition of *E. coli*. Also, 35% *v*/*v* of the EO combined with all 2, 4, and 8 mg/L of AMC resulted in total bacterial inhibition.

On the contrary, all combinations of AMC concentrations and *J. excelsa* EO showed total bacterial growth.

The combination of *M. barbata* and *E. globulus* EOs with AMC showed a synergistic effect against XDR *E. coli* (FICI = 0.50) when 25% of the EO was associated with 2 mg/L of AMC, as shown in Table 5. Additive action was also noted, with FCI values ranging from 0.60 to 0.90.

### 3.5. Effect of Associating Tetracycline, Gentamicin, and Ciprofloxacin with S. officinalis, J. excelsa, and M. barbata

In a control of the three EOs against MDR *C. coli* AX031, all the EOs showed almost total antimicrobial activity. All the combinations of TET, CIP, and GEN concentrations with the four tested Eos showed a total inhibition of *C. coli*.

The FICI values of EOs combined with antibiotics are shown in Table 6. A synergistic effect was highlighted (FICI = 0.50), and an additive action (FICI = 0.60–0.90) against MDR *C. coli*. A neutral effect (FICI = 1.25–1.45) when EOs were combined with the highest antibiotic concentration.

### 3.6. Effect of Associating Cefepime and Ciprofloxacin with M. barbata, J. excelsa, and E. globulus

All the combinations of the EOs with the antibiotic dilutions were ineffective and showed a total growth of *P. aeruginosa* CMUL 120 colonies.

### 3.7. Chemical Composition of the Essential Oils Tested in This Study

The EO of *M. barbata* is composed of various components, including pulegone, limonene, menthol, neomenthol, β-pinene, and piperitone, along with other ketones that contribute to its characteristic odor (Supplementary Table S1). The EO of *J. excelsa* was found to contain high amounts of α-pinene, followed by α-cedrol, limonene, and other compounds (Supplementary Table S2). The chemical analysis of *O. vulgare* EO revealed a total of 20 distinct components. The major constituents identified include carvacrol, p-cymene, and thymol, which collectively account for a significant proportion of the oil (Supplementary Table S3). Lastly, the EO of *E. globulus* exhibited a diverse composition with various identified compounds (Supplementary Table S4).

## 4. Discussion

Several studies have reported the antimicrobial activity of EOs and their bioactive compounds against Gram-negative and Gram-positive bacteria. However, data on the effectiveness of Lebanese EOs remain limited. Our findings highlight their notable antibacterial activity, particularly against MDR and XDR Gram-negative species. Antimicrobial resistance to bacterial pathogens is a critical issue associated with high morbidity and mortality rates [43]. The emergence of multidrug-resistant patterns in Gram-positive and Gram-negative bacteria poses significant challenges in treatment, often rendering conventional antibiotics ineffective or inadequate [43]. In recent years, researchers have explored alternative strategies to combat AMR, including the use of EOs or their bioactive constituents. The combinatorial approach, which leverages synergistic interactions between volatile EO compounds and antibiotics, has demonstrated enhanced antimicrobial efficacy [44].

Plant samples of *S. officinalis*, *E. globulus*, *M. barbata*, *O. vulgare*, *J. excelsa*, and *C. spinosa* were collected from the North Lebanon Region. EOs were extracted by hydro-distillation, and the resulting oils gave off a pleasant odor. Crude extracts of *C. spinosa* leaves were obtained using acetone and ethanol.

The antimicrobial activity results indicated that the oils exhibited significant effectiveness against bacterial strains with diverse resistance profiles. EOs showed a MIC range from 0.10% *v*/*v* to 50% *v*/*v* for the tested bacterial strains. *O. vulgare* revealed the highest antibacterial activity against *L. monocytogenes*, *E. coli*, and *Salmonella* spp., with a MIC of 0.10% *v*/*v*. The study conducted by Xiao et al. highlighted that oregano oil was the most effective agent in inhibiting *E. coli UTI 189*, with the lowest MIC (0.015% *v*/*v*) [45]. Recently, Pinto et al. found that oregano EO or carvacrol at 0.05% *v*/*v* could eradicate *S. Typhimurium* and *L. monocytogenes* at 0.5% *v*/*v* MIC. The differences in the oregano oil’s MIC against the strains can be due to the different resistance profiles of the bacteria. They also reported that the main antibacterial components were α-pinene, p-cymene, β-myrcene, and camphene. Nakas et al. identified carvacrol, γ-terpinene, p-cymene, and β-myrcene as the primary components of *O. vulgare* EO [46]. Our EO analysis revealed that carvacrol, p-cymene, and thymol were the main components. The different EO chemotypes, EO origin, and conditions for the EO application might have affected the composition of the EOs [47].

In this study, *O. vulgare* EO showed significant activity against *B*. *melitensis* at very low concentrations (0.20% *v*/*v*), followed by *J. excelsa* and *E. globulus* EOs. Studies on the bacterial effect of EOs against *B*. *melitensis* are limited compared to those conducted on other bacteria. Al-Mariri et al. confirmed the activity of *Origanum syriacum* against tetracycline-resistant *B. melitensis* [48]. Additionally, *C. spinosa* acetonic extracts (*C. spinosa*-ac) and *C. spinosa* ethanolic extracts (*C. spinosa*-etOH) from leaves showed the highest activity against *P. aeruginosa* among all tested plant extracts, with a MIC value of 12.5% *v*/*v*. The significant activity of *C. spinosa* extracts was against *L. monocytogenes*. On the other hand, the results showed modest activity of the extracts at high concentrations against *E. coli* and *Salmonella* spp. strains. The stronger antimicrobial activity of ethanolic extracts is likely attributed to the higher extraction efficiency of bioactive compounds in ethanol, resulting in higher concentrations of antimicrobial agents [49]. Gull et al. reported that *C. spinosa*-ac and *C. spinosa*-etOH extracts from roots exhibited the best antibacterial activity against *E. coli* and *Bacillus subtilis* [50]. In the recent research conducted by Al-Khafagi et al., *C. spinosa*-etOH had great antibacterial activity against *Helicobacter pylori* [51]. *C. spinosa* extracts exhibited variable degrees of antimicrobial activity related to its bioactive compounds, mainly polyphenols and flavonoids [52].

The findings regarding the combination of EOs with antibiotics are interesting. Several studies suggest that this approach significantly diminishes bacterial resistance and broadens the spectrum of antibiotic activity [53]. Moreover, this combination has been observed to lower the concentration of antibiotics required, consequently reducing their toxicity [53]. For instance, a study demonstrated that thymol, when combined with penicillin, showed synergistic activity against *E. coli*, reducing resistance and broadening the antibiotic’s effectiveness [54].

The results showed that the MIC of LEV decreased from 1 mg/L to 0.25 mg/L in the presence of *E. globulus* and *M. barbata* EOs, demonstrating a synergistic effect against the *A. baumannii* strain (FICI = 0.50). These combinations reduced the MIC values of LEV fourfold. The observed antibacterial activity of *E. globulus* EO is likely due to the presence of key bioactive compounds, such as p-cymene and eucalyptol (1,8-cineol), which are known for their antimicrobial properties. Iseppi et al. observed, using a checkerboard assay, the efficacity of oxacillin combined with *E. globulus* against methicillin-resistant *S. aureus* strains, with FICI values between 0.15 and 0.50 [53]. In a recent study, Santos et al. claimed the synergistic and additive effects after combining CRO and *Eucalyptus radiata* against *E. coli* strains isolated from meat products [55].

Despite the limited data available on the antimicrobial activity of *J. excelsa* and *M. barbata* EOs, El Omari et al. reported the efficacy of Lebanese *J. excelsa*, *M. barbata*, and *E. globulus* EOs against *Mycobacterium tuberculosis* and atypical mycobacteria [56]. Moreover, Alwan et al. showed a significant effect of *M. barbata* on *Candida albicans* [57]. In the same context, the present study revealed the antibacterial activity of *J. excelsa* EO against *Brucella melitensis*. According to El Omari et al., GC-MS analysis of *J. excelsa* EO identified 44 components, with high concentrations of α-pinene and α-cedrol, both known for their antimicrobial properties [56]. Similarly, *M. barbata* EO was characterized by a diverse composition, including pulegone, limonene, menthol, neomenthol, β-pinene, and piperitone, along with other ketone compounds [57].

The synergistic activity of LEV and AMI, when associated with *J. excelsa* EO against the *A. baumannii* strain, resulted in a reduction of MIC values of the antibiotics fourfold, with MIC values ranging from 1 mg/L to 0.25 mg/L and 8 mg/L to 2 mg/L, respectively. Furthermore, the additive action between AMI and *M. barbata* EO was obtained against the *P. aeruginosa* strain, with a decrease in MIC from 8 mg/L to 4 mg/L, and the reduction was twofold. Moreover, the decrease in the MIC of AMC was also associated with *M. barbata* EO against the *E. coli* strain from 8 mg/L to 2 mg/L. This synergistic combination reduced the MIC values of AMC fourfold.

In this research, *Salvia officinalis* demonstrated antimicrobial activity against *E. coli*, *P. aeruginosa*, *L. monocytogenes*, and *Salmonella* spp. This activity is primarily attributed to the presence of major components such as α-thujone, camphor, eucalyptol, α-humulene, and camphene in Lebanese *S. officinalis* EO [58]. A significant decrease in the MIC of TET, GEN, and CIP was observed when combined with *S. officinalis*, *J. excelsa*, and *M. barbata* EOs against the MDR *C. coli* strain. These synergistic combinations reduced the antibiotics’ MIC values fourfold. The association of GEN and TET with *S. officinalis* was also effective against *S. aureus* strains, as reported by Silva et al. [59]. Our data also showed no synergistic effect of CEF and CIP with EOs from *M. barbata*, *J. excelsa*, and *E. globulus* against *P. aeruginosa*. This lack of effect is related to the resistant nature of *P. aeruginosa* and its XDR phenotypic profile.

## 5. Conclusions

This study demonstrates that all the EOs tested exhibited antibacterial activity against the bacterial strains. Among the five EOs, *O. vulgare* showed the highest activity against *L. monocytogenes*, *E. coli*, *Salmonella* spp., and *B. melitensis* at very low concentrations. *C. spinosa* leaf extracts also displayed significant antibacterial activity, particularly against *L. monocytogenes*, with notable inhibition of *P. aeruginosa*, despite limited previous reports.

Notably, this research is the first to investigate the synergistic antibacterial effects of Lebanese *M. barbata* and *J. excelsa* EOs in combination with antibiotics against resistant bacterial strains. Given the growing concern about global antimicrobial resistance, the synergism observed between these EOs and antibiotics presents a promising strategy to address bacterial resistance.

However, while the findings indicate some significant differences in antibacterial activity, not all EOs exhibited consistent or statistically significant effectiveness across all bacterial strains. For instance, while *S. officinalis* and *J. excelsa* EOs showed significantly lower activities in some cases, others, such as *O. vulgare* and *E. globulus*, did not show significant differences in comparison. These nuances are required to understand the broader applicability of these EOs as potential antibiotic alternatives.

Although the results are promising, further investigation into the practical application of these EOs as alternatives to traditional antibiotics is essential. Considerations regarding their safety, efficacy, and clinical feasibility are paramount. Cytotoxicity studies and additional in vivo testing are necessary to assess better their potential for widespread clinical use.

## Figures and Tables

**Table 1:** Characteristics of the tested bacterial strains.

Bacterial Strain	Microbial Collection of the Lebanese University	Antimicrobial Resistance Profile	Phenotypic Profile
*Pseudomonas aeruginosa*	CMUL 122	TIC, TCC, PIP, TZP, GEN, AMI, TOB, CEF, OFL, MER, IMI, CIP, CAZ	Extensively drug-resistant (XDR) clinical strain
*Pseudomonas aeruginosa*	CMUL 120	TIC, TCC, PIP, TZP, GEN, AMI, NET, TOB, CEF, AZT, OFL, MER, IMI, CIP, CAZ	XDR clinical strain
*Acinetobacter baumannii*	CMUL 291	AMP, AMC, TIC, TCC, PIP, TZP, GEN, AMI, NET, TOB, CEF, AZT, MER, IMI, LEX, CXM, FOX, CFM, CTX, CAZ, ERT, TET	XDR clinical strain
*Salmonella* spp.	CMUL 216	AMP, AMC, TIC, PIP, CEF, CF, CRO, AZT, CXM, FOX, CTX, CAZ, TET, MIN	Extended-spectrum β-lactamase (ESBL)-producing clinical strain
*Campylobacter coli*	AX 031	AMP, AMC, CIP, TET	Multidrug-resistant (MDR) clinical strain
*Escherichia coli*	CMUL 260	AMP, AMC, TIC, TCC, PIP, TZP, GEN, TOB, CEF, AZT, CIP, LEX, CXM, FOX, CFM, CTX, CAZ, SXT	XDR clinical strain
*Escherichia coli*	CMUL 096	AMC, AMX, TIC, PIP, TZP, TOB, CEF, AZT, OFL, CXM, FOX, CTX, CAZ, NAL	XDR clinical strain
*Listeria monocytogenes*	AL 004	-	Wild-type clinical strain
*Brucella melitensis*	CMUL 057	SXT	Non-MDR clinical strain

Abbreviations: AMP, ampicillin; AMC, amoxicillin–clavulanic acid; AMX, amoxicillin; TIC, ticarcillin; TCC, ticarcillin-clavulanic acid; PIP, piperacillin; TZP, piperacillin-tazobactam; GEN, gentamicin; AMI, amikacin; NET, netilmicin; TOB, tobramycin; CEF, cefepime; CF, cephalothin; CRO, ceftriaxone; AZT, aztreonam; MER, meropenem; IMI, imipenem; CIP, ciprofloxacin; OFL, ofloxacin; LEX, cefalexin; CXM, cefuroxime; FOX, cefoxitin; CFM, cefixime; CTX, cefotaxime; CAZ, ceftazidime; ERT, ertapenem; TET, tetracycline; MIN, minocycline; SXT, trimethoprim/sulfamethoxazole; NAL, nalidixic acid; CMUL, Microbial Collection of the Lebanese University.

**Table 2:** Minimum inhibitory concentration of *Salvia officinalis*, *Eucalyptus globulus*, *Origanum vulgare*, and *Juniperus excelsa* EOs against clinical strains.

Bacterial Strains	Positive Control	Negative Control	Essential Oils (%)				Mean ± SD (%)
			*S. officinalis*	*E. globulus*	*O. vulgare*	*J. excelsa*	
*Escherichia coli* CMUL 260	+	−	6.25	1.56	0.10	ND	2.64 ± 3.21
*Escherichia coli* CMUL 096	+	−	3.13	25.00	0.20	>50.00	32.08 ± 46.61
*Salmonella* spp. CMUL 216	+	−	12.50	1.56	0.10	ND	4.72 ± 6.78
*Listeria monocytogenes* AL 004	+	−	25.00	0.78	0.10	ND	8.63 ± 14.18
*Pseudomonas aeruginosa* CMUL 122	+	−	50.00	50.00	25.00	ND	41.67 ± 14.43
*Brucella melitensis* CMUL 057	+	−	>50.00	12.50	0.20	6.25	29.74 ± 47.11
Mean ± SD (%)			32.81 ± 37.07	15.23 ± 19.46	4.28 ± 10.15	53.13 ± 66.29	

Positive control: wells containing bacterial inoculum (10⁶ CFU/mL) in BHI broth without EOs; negative control: wells containing only BHI broth without bacterial inoculum; −: no bacterial growth; +: bacterial growth; ND: not determined; SD: standard deviation. For statistical analysis: values reported as >50 (indicating no antibacterial activity) were considered as 100, as the highest tested concentration was 100%.

**Table 3:** Fractional inhibitory concentration index (FICI) of *Micromeria barbata* EO tested in combination with amikacin against extensively drug-resistant (XDR) *Pseudomonas aeruginosa* strain.

MIC of *M. barbata* (mg/L)	MIC of Amikacin (mg/L)	MIC of *M. barbata* Combined with Amikacin (mg/L)	MIC of Amikacin Combined with *M. barbata* (mg/L)	FICI
>1,000,000	>8	45,000	8	1.45
			4	0.95
			2	0.70
		35,000	8	1.35
			4	0.85

All experiments were conducted in triplicate with consistent results.

**Table 4:** Fractional inhibitory concentration index (FICI) of *Juniperus excelsa*, *Micromeria barbata*, and *Eucalyptus globulus* EOs tested in combination with levofloxacin and amikacin against extensively drug-resistant (XDR) *A. baumannii* strain.

**(A)**			
**Levofloxacin (mg/L)**	**1**	**0.5**	**0.25**
***E. globulus*** **or *J. excelsa* EO (mg/L)**			
45,000	1.45	0.95	0.70
35,000	1.35	0.85	0.60
25,000	1.25	0.75	0.50
**(B)**			
**Amikacin (mg/L)**	**8**	**4**	**2**
***J. excelsa* EO (mg/L)**			
45,000	1.45	0.95	0.70
35,000	1.35	0.85	0.60
25,000	1.25	0.75	0.50
**(C)**			
**Amikacin (mg/L)**	**2**		
***E. globulus*** **EO (mg/L)**			
45,000	0.70		
35,000	0.60		
25,000	0.50		
**(D)**			
**Levofloxacin (mg/L)**	**0.5**		
***M. barbata* EO (mg/L)**			
3900	0.50		
1000	0.50		

All experiments were conducted in triplicate with consistent results.

**Table 5:** Fractional inhibitory concentration index (FICI) of *Micromeria barbata* and *Eucalyptus globulus* EOs tested in combination with amoxicillin–clavulanic acid against extensively drug-resistant (XDR) *Escherichia coli* strain.

MIC of *M. barbata* or *E. globulus* EO (mg/L)	MIC of Amoxicillin–Clavulanic Acid (mg/L)	MIC of *M. barbata* EO Combined with Amoxicillin–Clavulanic acid (mg/L)	MIC of Amoxicillin–Clavulanic Acid Combined with *M. barbata* EO (mg/L)	FICI	MIC of Amoxicillin–Clavulanic Acid Combined with *E. globulus* EO (mg/L)	FICI
>1,000,000	>8	450,000	2	0.70	8	1.45
					4	0.90
					2	0.70
		350,000	8	1.35	8	1.35
			4	0.85	4	0.85
			2	0.60	2	0.60
		250,000	2	0.50	8	1.25

All experiments were conducted in triplicate with consistent results.

**Table 6:** Fractional inhibitory concentration index (FICI) of *Juniperus excelsa*, *Micromeria barbata*, and *Salvia officinalis* EOs tested in combination with ciprofloxacin, gentamicin, and tetracycline against multidrug-resistant *Campylobacter coli* strain.

**(A)**			
**Ciprofloxacin (mg/L)**	**0.5**	**0.25**	**0.125**
***J. excelsa*, or *M. barbata*, or** ***S. officinalis* EOs (mg/L)**			
45,000	1.45	0.90	0.70
35,000	1.35	0.80	0.60
25,000	1.25	0.70	0.50
**(B)**			
**Gentamicin or Tetracycline (mg/L)**	**2**	**1**	**0.5**
***J. excelsa*, or *M. barbata*, or** ***S. officinalis* EOs (mg/L)**			
45,000	1.45	0.90	0.70
35,000	1.35	0.80	0.60
25,000	1.25	0.70	0.50

All experiments were conducted in triplicate with consistent results.

## Data Availability

The original contributions presented in this study are included in the article/Supplementary Materials. Further inquiries can be directed to the corresponding author.

## Supplementary Materials

The following supporting information can be downloaded at https://www.mdpi.com/article/10.3390/pathogens14040348/s1, Table S1. Chemical composition of the essential oil of *Micromeria barbata*. Table S2. Chemical composition of the essential oil of *Juniperus excelsa.* Table S3. Chemical composition of the essential oil of *Origanum vulgare.* Table S4. Chemical composition of the essential oil of *Eucalyptus globulus*.
