# Supplementary material for: Synergistic Antibacterial Effects of Plant Extracts and Essential Oils Against Drug-Resistant Bacteria of Clinical Interest
Source: Pathogens. 2025 Apr 4;14(4):348. doi: 10.3390/pathogens14040348 (PMC12030331; doi:10.3390/pathogens14040348)
Supplement: Supplementary file 1 [file pathogens-14-00348-s001.zip › pathogens-3502055-supplementary.pdf]

Supplementary tables

Table S1. Chemical composition of the essential oil of *Micromeria barbata*.

Compound name	% Area
α-Pinene	1.76
Camphene	0.10
β-Pinene	3.29
β-Myrcene	1.56
3-octanol	1.16
Limonene	16.59
Ocimene	0.17
Terpinolene	0.10
Menthone	0.16
Neomenthol	12.37
Menthol	6.19
Pulegone	20.19
Piperitone	4.22
Menthyl acetate	0.35
Isomenthol acetate	0.89
Cadinene	1.06
Cedrol	0.49

Table S2. Chemical composition of the essential oil of *Juniperus excelsa.*

Compound name	% Area
α-Pinene	76.91
α-Cedrol	3.68
Limonene	2.45
β-Pinene	1.06
Terpinene	0.86
β-Cedrene	0.71
p-Cymene	0.64

Table S3. Chemical composition of the essential oil of *Origanum vulgare.*

Compound name	% Area
Carvacrol	37.81%
p-Cymene	19.44%
Thymol	18.06%
γ-Terpinene	10.75%
Vinyl amyl carbinol	0.50%
Limonene	0.73%
α- Thujene	1.44%
α- Pinene	0.70%
β- Pinene	0.22%
(E)-β-Ocimene	0.16%
α- Terpinene	3.37%
4-Thujanol, Stereois	0.16%
δ-2-Carene	0.15%
2-Bornanol	0.08%
Terpinen-4-ol	0.82%
Myrcene	2.85%
Ethyl hexanol	0.76%
α- Phellandrene	0.47%
Caryophyllene	0.66%
Camphene	0.09%

Table S4. Chemical composition of the essential oil of *Eucalyptus globulus*.

Compound name	% Area
p-Cymene	35.44
Eucalyptol	15.60
Spathulenol	12.29
Sabinene	7.63
Menthyl acetate	4.99
Terpinene	1.45
α-Pinene	0.54
